# High Levels of Hemoglobin Promote Carotid Adventitial Vasa Vasorum Neoangiogenesis in Chronic Kidney Disease

**DOI:** 10.1155/2017/3795142

**Published:** 2017-01-04

**Authors:** Maria Vittoria Arcidiacono, Montserrat Martinez-Alonso, Montserrat Belart, Ana Vilar, Marisa Martín, Lourdes Craver, Àngels Betriu, Dídac Mauricio, José Manuel Valdivielso, Elvira Fernández, Mercè Borràs

**Affiliations:** ^1^Department of Morphology, Surgery and Experimental Medicine and LTTA Centre, University of Ferrara, Ferrara, Italy; ^2^Vascular and Renal Translational Research Group, Institut de Recerca Biomedica de Lleida (IRBLleida), Lleida, Spain; ^3^Unitat de Detecció i Tractament de Malalties Aterotrombòtiques, Hospital Universitari Arnau de Vilanova, Lleida, Spain; ^4^Statistics Department, IRBLleida, Lleida, Spain; ^5^Sistemes Renals, Lleida, Spain; ^6^Department of Nephrology, Hospital Universitari Arnau de Vilanova, Lleida, Spain; ^7^Department of Endocrinology and Nutrition, CIBER of Diabetes and Associated Metabolic Diseases, Hospital Universitari Germans Trias i Pujol, Badalona, Spain

## Abstract

Chronic kidney disease (CKD) patients, characterized by traditional and nontraditional risk factors, are prone to develop atheromatosis and thus cardiovascular events and mortality. The angiogenesis of the adventitial vasa vasorum (aVV) surrounding the carotid has been described as the atheromatosis initiator. Therefore, the aim of the study was to (1) evaluate if the carotid aVV in CKD patients increases in comparison to its physiological value of healthy patients; (2) explore which traditional or nontraditional risk factor including inflammation, bone and mineral metabolism, and anemia could be related to the aVV angiogenesis. CKD patients without previous cardiovascular events (44, stages 3-4; 37, stage 5D) and 65 healthy subjects were compared. The carotid aVV and the intima-media thickness (cIMT) were evaluated by ultrasound. CKD patients at stages 3-4 showed higher aVV of the right carotid artery even after adjusting for age. Importantly, a multiple linear regression model showed hemoglobin levels > 12.5 g/dL as the factor for an estimated higher aVV of the right carotid artery. In conclusion, the association of hemoglobin with higher aVV could suggest the role of high hemoglobin in the higher incidence of adverse cardiovascular outcomes in CKD patients.

## 1. Introduction

Chronic kidney disease (CKD) has been reported as a major risk factor for cardiovascular (CV) disease [1]. Indeed, CKD patients at all stages demonstrate a marked increase in the incidence of CV events and CV disease mortality in comparison with age- and sex-matched subjects of the general population [2] and, in particular, in dialysis patients, CV mortality rate is 10 times higher than that for the general population [3, 4]. This high incidence is due to the fact that not only are patients with CKD affected by traditional risk factors including hypertension, diabetes, dyslipidemia, and smoking [5], but they are also affected by nontraditional risk factors including inflammation, altered bone and mineral metabolism, anemia, and albuminuria. Both traditional [6, 7] and nontraditional risk factors [8–11] are associated with the impairment of the endothelial function and the inflammatory response [12], key steps in the development of atherosclerosis, plaque progression, and a higher risk of CV events.

Challenging the common belief that subclinical endothelial dysfunction has been considered the earliest step in the chronic inflammation of the vessel wall, studies by Herrmann et al. demonstrated conclusively that, during pathological conditions, increases in the density of adventitial vasa vasorum (VV), the plexus of physiological microvessels surrounding the adventitial layer, precede endothelial dysfunction [13].

Inflammation has been strongly related to VV neoangiogenesis [14]. Indeed, inflammation is associated with the recruitment of circulating inflammatory cells as neutrophils, macrophages, and lymphocytes which secrete proangiogenic factors including cytokines and the well characterized signaling molecule Vascular Endothelial Growth Factor (VEGF) [15, 16]. Together with inflammation, elevated levels of parathyroid hormone (PTH), lower levels of vitamin D, and anemia, all common features of CKD, have been recognized as proangiogenic stimuli. Specifically, recent experimental animal studies associated PTH treatment with increased angiogenesis in both the ischemic brain and the infarcted heart [17, 18]. Equally important in the angiogenesis process is the evidence of the involvement of vitamin D. Oikawa et al. showed that active vitamin D, 1*α*,25-dihydroxyvitamin D_3_ (1,25D), was highly effective in inhibiting angiogenesis in a dose-dependent manner in chick embryo chorioallantoic membranes [19]. Consequently, while several lines of evidence support the efficacy of 1,25D to suppress neoangiogenesis through strong anti-VEGF properties [20–22], others demonstrated that calcitriol exerts antiangiogenic properties in a VEGF-independent manner. Indeed, in the retina, calcitriol suppresses neovascularization through the inhibition of the endothelial cells proliferation and sprouting [23]. Additionally, a third feature of CKD that could be involved in angiogenesis and therefore in the increases of the adventitial VV is anemia. In a study of 59 patients with nonmalignant cancers, 23 patients with renal anemia exhibited significantly higher levels of VEGF in comparison to those of normemic patients [24]. Furthermore, in a CKD population study including patients at stages 3b-4, the adjusted linear inverse association of the flow-mediated dilatation with hemoglobin (Hb) in a range between 8.5 and 14.5 g/dL, demonstrated that (Hb) per se affects the endothelial function regulation which could likely determine the VV neovascularization [25, 26].

Since, to our knowledge, there are no reports describing the adventitial VV density in patients with renal disease nor the pathophysiology of adventitial VV density in this population, the current study was designed to compare the carotid adventitial VV density in CKD patients with the physiological carotid adventitial VV density in healthy volunteers with none of the classical risk factors. Moreover, since, inflammation, high levels of PTH, vitamin D deficiency, and anemia are generally observed features of CKD, we examined the correlation of these variables, including VEGF, with the carotid adventitial VV content in order to evaluate their role in increasing the carotid adventitial VV, therefore aggravating CV disease (CVD), in both dialysis and nondialysis-dependent CKD patients.

## 2. Material and Methods

### 2.1. Study Subjects

In this single-centre, cross-sectional study, 81 patients with CKD (53% males; median age 60 [53.0; 66.0]) were enrolled as the case group according to a previous selection of 104 CKD patients: patients were withdrawn from the study because of poor or overly strong echogenicity or because of poor or inaccessible venous access. Further exclusion criteria included the following: (1) they experienced previous cardiovascular events such as coronary heart disease, cerebrovascular disease, and peripheral vascular disease; (2) they met the exclusion criteria for the administration of the contrast agent including (a) recent cardiac instability, (b) recent (<7 days) coronary intervention, (c) class III or IV heart failure, (d) severe pulmonary hypertension, and (e) allergic reaction to sulphur hexafluoride, the gas contained in the contrast agent. Specifically, 44 patients belonged to CKD stages 3-4, while 37 belonged to CKD stage 5D (28 hemodialysis patients and 9 peritoneal dialysis patients). Sixty-five subjects with none of the classical risk factors for cardiovascular disease (46% male; median age 49 [41; 57]) were enrolled as control group. The age range was between 30 and 70 years.

The study protocol was approved by the ethical committee at the University Hospital Arnau de Vilanova (HUAV, Lleida, Spain). All subjects signed an informed consent prior to morphometric parameter acquisition and blood drawing after overnight fasting.

### 2.2. Morphometric and Biochemical Parameters

Morphometric body parameters such as weight (kilograms) and height (meters) were measured with a digital weight scale equipped with a stadiometer. Body mass index (BMI) was calculated as weight divided by the square of height. The waist circumference (WC) was measured in the umbilicus level (cm). While patients were seated and had rested for ten minutes, systolic and diastolic blood pressure (SBP and DBP) were obtained with an automated oscillometer (Omron HEM-705CP) and calculated as the average of three independent measurements. Serum levels of glucose, total cholesterol (cholesterol), HDL and LDL cholesterol, triglycerides (TG), and ultrasensitive C-reactive protein (CRP), creatinine clearance (CC), calcium (Ca), phosphorous (P), PTH, albumin (Alb), ferritin, hemoglobin (Hb), fibrinogen, and leukocytes were obtained using the standard methods of the laboratory of Clinical Biochemistry at the HUAV. For serum VEGF, the Human VEGF ELISA Kit was used (R&D System, DVE00). Serum levels of 25-hydroxyvitamin D (25D) were measured using the Chemiluminescence Immunoassay on the LIAISON XL Analyzer (DiaSorin), while serum 1,25D levels were determined using a radioreceptor assay (Gamma-B dihydroxyvitamin D, IDS Hybritec®).

### 2.3. Standard and Contrast-Enhanced Carotid Ultrasound

All subjects underwent a B-mode ultrasound examination of the extracranial carotid arteries (CA). For the measurements of carotid intima-media thickness (cIMT; mm) of the far wall of the common carotid artery (CCA) 1 cm proximal to the bifurcation and for the evaluation of atheromatous plaque presence, a prior axial exploration was followed by a longitudinal exploration. Carotid plaque was defined as a cIMT > 1.5 mm or as a focal thickening overpassing into the arterial lumen by at least 50% of the surrounding cIMT value [27, 28]. Then, subjects underwent the contrast-enhanced ultrasound (CEUS) imaging procedure using the contrast agent SonoVue (Bracco Spa, Milan, Italy). The contrast agent was prepared as recommended on the manufacturing data sheet, and, for each CA explored, a 2.5 mL contrast agent bolus, followed by 10 mL of a saline, was injected in the antecubital vein (20-gauge needle). This chosen dose was the lowest dose adequate to reach a strong and clear signal that suffice to obtain statistically significant differences between the adventitial VV signal in control subjects and CKD patients based on the previous differences obtained between type 2 diabetic patients with or without retinopathy and control subjects [29]. The CEUS imaging was performed with a Siemens Sequoia 512 using the 15L8W linear array probe (7 Mhz) with a low mechanical index of 0.18. This device is equipped with Cadence contrast pulse sequencing (CPS) technology able to determine a high sensitivity and specificity of contrast agent detection. All the videos were analyzed using the Siemens software Syngo. Adventitial VV content was measured as previously described [30]. Videos in which signals (clipping artefacts) were observed in the far arterial wall before contrast injection were excluded from reading. Moreover, additional reasons for reading exclusion were as follows: (1) the contrast agent which disappeared rapidly, thus impeding proper visualization or (2) the presence of an ultrasound shadow that impeded the reading in the area under analysis.

Since the optimal cIMT measurement is obtained on the far wall and because our applied method elicits strong clipping artefacts in the near wall [31], in accordance with other reports exploring the adventitial and intraplaque VV [32–34], the VV signal was measured in the far wall as well. Importantly, although the far wall could be liable to imaging artefacts due to nonlinear propagation [35], there is a licit expectation that these artefacts are similar for healthy controls and CKD patients.

### 2.4. Statistical Analysis

Descriptive statistics of mean (standard deviation) or median [interquartile range] were estimated for quantitative variables with a normal or nonnormal distribution, respectively, while, for qualitative variables, absolute and relative frequencies were used. Normal distribution was analyzed using the Shapiro-Wilks test.

The significance of the differences in quantitative variables between groups (control, CKD stages 3-4, and CKD stage 5D) was assessed by analysis of variance or Kruskal-Wallis test depending on their normal distribution. The significance of the differences in qualitative variables between the three studied groups was assessed by Chi-squared test or Fisher's exact test in case of any expected frequency of the corresponding contingency table lower than 5. In case of significant differences, multiple testing adjusted pairwise comparisons were performed using the Tukey or Benjamini-Hochberg method according to a normal or not normal distribution. Linear regression models were fitted to each measure of cIMT and carotid adventitial VV in order to assess differences between groups considering or not age as angiogenic factor. The most adequate Box-Cox transformation was applied in case of nonnormally distributed residuals from multivariable regression models. Within the CKD population, monotonic relationships of the right and left carotid adventitial VV with quantitative characteristics were assessed by using Spearman's rank correlation coefficients. In case of significant association, age-adjusted linear regression models were fitted. Associations of the right and the left carotid adventitial VV with dichotomous characteristics of each CKD studied stage were assessed by Mann–Whitney *U* test (Kruskal-Wallis test for smoking status). An adjusted linear regression model was fitted to the right carotid adventitial VV mean, including the assessment of the significant interactions. Only interactions or main effects with a significant contribution to the final model according to the likelihood ratio test were included. All the studied variables (see Tables 1 and 5) were assessed for significant contribution into the model. A significance level of 0.05 was fixed previously to the statistical analysis of the study data with the freeware statistical software R [36]. A figure to illustrate the estimated right carotid adventitial VV mean and its confidence intervals was performed to show the relationship with hemoglobin, once adjusted by all the significant variables and their interactions.

## 3. Results

Baseline characteristics of the study populations, controls (C) and patients with chronic kidney disease at stages 3-4 and 5D, are shown in Table 1. According to disease characteristics, subjects affected by CKD were significantly older than subjects of the control group (*p* = 0.001, CKD 3-4 versus C; *p* < 0.001, CKD 5D versus C). Moreover, as expected from the inclusion criteria of the selected control population with none of the classical risk factors for cardiovascular disease and according to the disease characteristics, body mass index (BMI, *p* = 0.013, CKD 3-4 versus C), waist circumference (WC, *p* < 0.001, CKD 3-4 versus C; *p* = 0.017, CKD 5D versus C), serum glucose (*p* = 0.004, CKD 3-4 versus C), triglycerides (TG, *p* < 0.001, both CKD groups versus C), systolic blood pressure (SBP, *p* < 0.001, both CKD groups versus C), and diastolic blood pressure (DBP, *p* < 0.001, CKD 3-4 versus C), as well as serum C-reactive protein (CRP, *p* < 0.001, CKD 5D versus C), were significantly higher in patients with CKD. Additionally, serum 25-hydroxyvitamin D (25D) levels and LDL cholesterol were lower in the CKD 5D group than in the control group (*p* < 0.001 and *p* = 0.018, resp.). On the other hand, serum VEGF levels were significantly higher in both CKD groups than in control subjects (*p* = 0.004 and *p* = 0.013). Finally, dialysis patients showed, in comparison with nondialysis CKD patients, a significantly higher CRP (*p* = 0.001) and lower levels of 25D (*p* < 0.001).

The analysis of the cIMT and of the carotid adventitial VV showed that, after adjusting by the natural atherogenic factor age, CKD 5D patients had a higher cIMT only in the right carotid artery (Table 2: *p* = 0.002 and *p* = 0.043, CKD 5D versus C and CKD 3-4, resp.), while the adventitial VV were significantly higher only in the right carotid artery of CKD patients at stages 3-4 (Table 2: *p* = 0.007, CKD 3-4 versus C)

Tables 3 and 4 show the unadjusted associations between the adventitial VV and biochemical/anthropometric and clinical variables known to be potentially involved in the development of atheromatosis. As shown in Table 3, in CKD patients at stages 3-4, the adventitial VV of both carotid arteries did not correlate with any of the studied variables. On the other hand, in patients at CKD stage 5D, the right adventitial VV positively and monotonically associated with serum glucose and HDL cholesterol (*p* = 0.0089 and *p* = 0.0073, resp.), while the left adventitial VV negatively associated with serum calcium (*p* = 0.0072) and positively associated with albumin (*p* = 0.0392). Nevertheless, when an age-adjusted linear regression model was applied, significantly higher right adventitial VV were identified only for the second and the third tertile of glucose levels (90–104 and 105–227 mL/dL; *p* = 0.0241 and *p* = 0.0099, resp.). This association is consistent with the right adventitial VV significant association with diabetes in dialysis patients (Table 4: *p* = 0.038).

Since this was the main goal of the study, we further explored if there were differences between the two CKD groups in any of the characteristic variables of the disease (Table 5). As a consequence of the disease stages, phosphorus (P), ferritin, and PTH were higher in dialysis patients (*p* < 0.001; *p* = 0.001; *p* < 0.001, resp.). On the other hand, serum calcium (Ca), 1,25D, and albumin were lower in dialysis patients (*p* = 0.001; *p* < 0.001 and *p* < 0.006, resp.). As expected, CKD patients on dialysis, due to the high incidence of anemia, required more treatment with erythropoiesis stimulating agents (ESA) and/or iron than nondialysis patients (*p* < 0.001 and *p* = 0.001, resp.), while the level of hemoglobin was lower than that in the nondialysis patients (*p* < 0.001).

**Figure 1:**
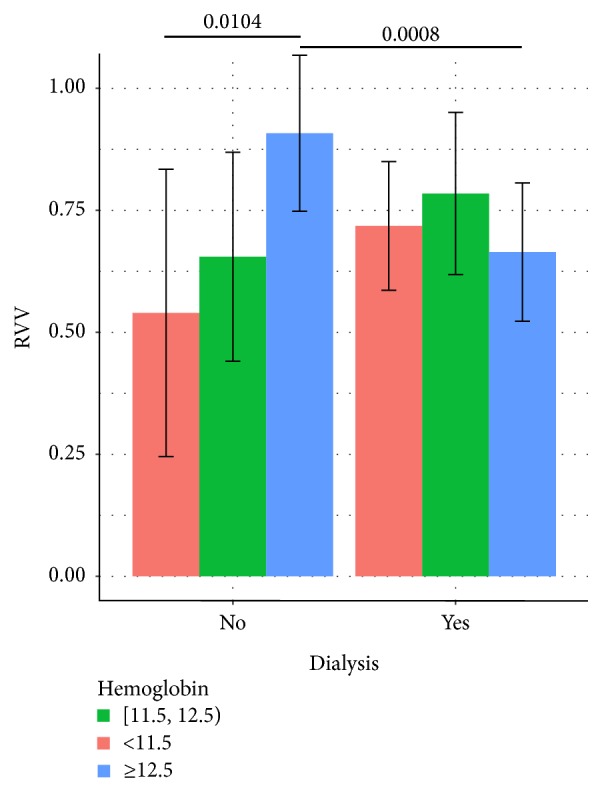
Effect of hemoglobin and dialysis on the right carotid adventitial VV. Hemoglobin at levels of 12.5 g/dL or above determines higher levels of the right carotid adventitial VV (RVV). Bars and error bars represent the mean and the 95% CI of the RVV.

Finally, an adjusted linear regression model was fitted in order to explore how the variables involved in angiogenesis and CKD stage could contribute to the increase in the adventitial VV content (Table 6). Specifically, significant differences in the carotid adventitial VV were determined by the hemoglobin levels depending on whether or not patients were on dialysis. As shown in Figure 1, the adjusted mean right VV (RVV) was estimated to be statistically higher in CKD nondialysis patients with hemoglobin levels of 12.5 mg/dL or above than in CKD nondialysis patients with hemoglobin levels below 12.5 mg/dL (0.91 versus 0.54; *p* = 0.0104). Furthermore, for hemoglobin levels of 12.5 mg/dL or above, CKD nondialysis patients showed an estimated mean RVV higher than that for CKD dialysis patients (0.91 versus 0.66; *p* = 0.0008).

In addition, two more variables significantly contributed to the model: 1,25D and P (Table 6). Specifically, the levels of 1,25D were related to the right carotid adventitial VV only for those patients with low P levels (below its median value). Consequently, the estimated mean RVV was statistically different for CKD patients with low 1,25D and P below 4 mg/dL compared to that of the patients with P levels above 4 mg/dL (0.54 versus 0.36; *p* = 0.039).

Furthermore, since ESA treatment is a recognized angiogenic factor and it is associated with being on dialysis, the difference between ESA users and nonusers in both CKD populations was explored: no differences in the right VV associated with the use of ESA. Moreover, no significant interaction between ESA use and hemoglobin levels associated with the right carotid adventitial VV (data not shown).

## 4. Discussion

This study demonstrated that only the right carotid artery (CA) is characterized by higher cIMT and adventitial VV in CKD patients than in control subjects. In addition, of high relevance from the nephrologist's point of view, this study identified hemoglobin as the factor that, at levels of 12.5 g/dL or above, determines the highest estimated adventitial VV of the right carotid artery in CKD patients at stages 3-4.

Specifically, in our study population, after adjusting for age, a natural atherogenic factor, the highest cIMT, was observed only in the right carotid artery of CKD patients undergoing dialysis, while the highest adventitial VV content was observed in CKD patients at stages 3-4 suggesting the carotid adventitial VV neoangiogenesis as the earliest step in the intima-media thickening. The difference in cIMT observed in the right carotid artery, but not in the left, is in accordance with several clinical studies in which asymmetrical differences between the left and the right cIMT are modulated by altered biochemical and hemodynamic parameters [37, 38]. For instance, Chaubey et al.'s findings demonstrated that the difference in cIMT, higher in the left than in the right carotid artery in normotensive subjects, is attenuated when considering subjects with a mean blood pressure higher than 90 mmHg. Moreover, divergent values of the right and the left cIMT could be explained by the fact that the left cIMT thickens every ten years after the age of 35, while the same trend occurs in the right cIMT ten years later and it is correlated with hemodynamic parameters as observed by Luo and colleagues [39]. Importantly, our results demonstrated that, at earlier stages of CKD, the adventitial VV content is also higher only in the right carotid artery in comparison with that in the control population, probably due to similar hemodynamic changes such as shear stress. Previous studies in healthy individuals without classical risk factors for atheromatosis demonstrated that the left carotid adventitial VV correlated with age and the left cIMT, explaining in part the earliest appearance of atheromatosis lesions in the left carotid artery [30, 40]. Taken together, these results underline the importance of differentiating between the left and the right carotid artery not only in control subjects or in the general population but also in patients with known cardiovascular risk factors which could determine heterogeneous atheromatosis in different territories of the vascular bed. Indeed, although the differences in the two carotid arteries are scarcely investigated, some works reported how local factors, mainly the geometry of the carotid artery, may play a role in the heterogeneous atherosclerotic lesion localization due to different blood flow patterns that influence changes in the shear stress [41, 42].

Unfortunately, the role of the carotid adventitial VV in CKD patients and the factors that modulate its content are still unknown. Consequently, the aim of this study was to explore not only the content of adventitial VV but also how angiogenic factors that are modified in CKD could have been involved in its increase. Therefore, since cIMT and adventitial VV were higher in the right carotid artery of the overall CKD population in parallel with higher levels of inflammation, TG, glucose, and blood pressure, as well as with lower levels of vitamin D, we evaluated the relationship between these variables and VV content in both dialysis and nondialysis patients. The unadjusted analysis revealed that while the VV of CKD patients at stages 3-4 did not correlate with any of the studied biochemical or anthropometric parameters, all of which are highly involved in the development of atheromatosis, the VV of patients on dialysis was positively associated only with serum glucose levels at the second and third tertile (90–104 and 105–227 mg/dL) and positively associated with diabetes. This result is in accordance with the increase of carotid adventitial VV in type 2 diabetic patients with hyperglycemia even in absence of retinopathy [29]. The lack of association of VV with glucose in CKD patients at stages 3-4 characterized by higher glucose levels suggests that VV increase is driven by distinct factors according to the stage of the disease. Indeed, previous studies have demonstrated that while, in the general population and CKD patients at stage 3, there was a significant interaction between smoking and TG which were independently associated with atheromatosis [43, 44], in patients at advanced stages, this association was lost. Similar results, depending on the stages of the disease and the interaction between biochemical and life habit risk factors, were observed for CRP which only at CKD stages 4-5 was associated with smoking, high phosphate, and atheromatosis only at the highest tertile [44, 45]. Moreover, these results are in agreement with Sampson and colleagues' study that proved no remarkable variations of VV content with respect to the range of measurements of systolic and diastolic blood pressure [33]. On the contrary, our results do not coincide with experimental evidence that supports a role of LPS-induced inflammation and high TG in the increase of adventitial VV measured by CEUS and histological analysis in rabbits with atherosclerosis [46]. Indeed, although CKD patients are characterized by elevated CRP and TG levels, this is a transversal cohort study in which neither time nor levels of exposure of the artery wall to biochemical parameters have been taken into account. Therefore, in accordance with the demonstration that the atheromatous process is driven by specific risk factors according to the CKD stage [44], an adjusted linear regression model was used to identify the variables that could independently predict the increase of the carotid adventitial VV accordingly whether or not patients were on dialysis. This analysis demonstrated that Hb levels interact with the dialysis status. Specifically, in CKD patients at stages 3-4, hemoglobin higher than 12.5 g/dL is a predictor of an estimated higher adventitial VV of the right artery. Although a high percentage of CKD patients were under ESA treatment, especially those being on dialysis, no difference in the adventitial VV content was associated with the use of ESA. Therefore, the hemoglobin status in our CKD nondialysis study population patients could be considered a natural angiogenic mediator. This is of high relevance in nephrology, since anemia greatly influences cardiovascular outcomes. For instance, in Vlagopoulos et al.'s study, in which four community-based trials were pooled, it was reported that, in CKD patients with anemia, the risk of CVD was 1.7-fold higher [47]. On the other hand, intervention with ESA for the correction of renal anemia might increase occurrence of CVD. In fact, in the CREATE study, the complete correction of anemia did not delay the onset of cardiovascular events [48]. Moreover, in the CHOIR study, target Hb level of 13.5 g/dL in nondialysis CKD patients were associated with increased risk for death, myocardial infarction, and cardiovascular events [48, 49]. Important to support our results is the demonstration by Yilmanz et al. that nondiabetic CKD patients at stages 3-4, not using erythropoietin-based agents, showed a reduction of the flow-mediated dilatation for hemoglobin levels higher that 11.6 g/dL. This is in contrast with the finding of the prospective and observational Dialysis Outcomes and Practice Patterns Study in which the authors showed that the natural increase of Hb to concentration >12 g/dL in hemodialysis patients did not associate with increased mortality, suggesting that high Hb levels per se are not harmful for CKD patients [50].

The interaction of 1,25D and P in the estimation of the adventitial VV of the right carotid artery is intriguing. Indeed, calcitriol is inversely correlated with the carotid adventitial VV and is statistically different only for P levels below its median value (4 mg/dL). This apparent contradiction could be due to the vitamin D supplementation, paricalcitol treatment, and also the P-binding treatment that were not taken into account in the present study.

As has been noted, this limitation of study could be related to the administration of paricalcitol which could have a direct effect on angiogenesis [51], inflammation [52] and bone mineral metabolism, and above all PTH [53], while the serum levels of active vitamin D cannot be measured except for the calcitriol form. Further studies on a larger sample size are needed to clarify the role of 1,25D and P on VV angiogenesis. In addition to the low number of patients, the present study is a cross-sectional study, the reason why there is no indication on how the factors taken into account contributed to a temporal relationship between exposure to these risk factors and the increase of VV. Therefore, a future study should be designed to evaluate the outcomes over time, also taking into account the time of exposure to treatment. Moreover, according to our results, the lack of hemoglobin levels and bone mineral metabolism in the control population impeded the evaluation of the physiological association of these parameters with neoangiogenesis in a healthy population.

Despite these limitations, this study not only confirms the strength of the use of CEUS in measuring adventitial VV as a predictor of the atheromatous process based on the fact that there is a high object evaluation of the imaging (high intraobserver reproducibility) but also is a further confirmation of the importance of separately evaluating the left and the right carotid artery. Moreover, this study, to our knowledge, is the first measuring the carotid adventitial VV in a CKD population and the first indicating that Hb levels and the interaction of 1,25D and P could be associated with the carotid adventitial VV in this population.

In conclusion, this work suggests that, in CKD patients at stages 3-4, hemoglobin levels higher than 12.5 g/dL might be a cause for the increased adventitial VV of the right CA that consequently could increase the right cIMT. Therefore, it could be speculated that the higher incidence of atheromatous disease in CKD patients could be driven by a higher incidence of microangiopathy of the common carotid wall driven by the hemoglobin status. Indeed, this could explain why Hb levels as in the CHOIR study associated with higher cardiovascular events and risk of death [49]. Consequently, as previously specified, a longitudinal study with a higher number of patients is required to evaluate the relationship of Hb levels and the carotid adventitial VV in the onset of the process of atheromatosis and cardiovascular events in CKD patients.

## Figures and Tables

**Table 1:** Baseline characteristics of the study populations.

	Control (*n* = 65)	CKD 3-4 (*n* = 44)	CKD 5D (*n* = 37)	*p* overall	*p* (C versus CKD 3-4)	*p* (C versus 5D)	*p* (CKD 3-4 versus 5D)
Age (years)	49 [41.0; 57.0]	59.5 [52.8; 65.0]	60.0 [53.0; 68.0]	*<0.001*	0.001	<0.001	0.214
Sex							
Man	30 (46.2%)	27 (61.4%)	16 (43.2%)	0.189^#^			
Women	35 (53.8%)	17 (38.6%)	21 (56.8%)				
BMI (Kg/m^2^)	24.8 [22.9,26.3]	27.8 [24.0; 30.7]	25.9 [23.7; 27.9]	*0.010*	0.013	0.130	0.151
WC (cm)^**∗**^	88.6 ± 9.29	97.3 ± 12.4	94.8 ± 10.0	*<0.001*	<0.001	0.017	0.533
Glucose (mg/dL)	91.0 [87.0; 96.0]	100 [90.0; 113]	93.0 [84.0; 102]	*0.009*	0.004	0.928	0.088
Total cholesterol (mg/dL)	183 [162; 195]	181 [165; 206]	172 [148; 198]	*0.353* ^*#*^			
HDL cholesterol (mg/dL)	54.0 [45.0; 63.0]	50.0 [44.0; 61.0]	48.0 [41.0; 53.0]	*0.087* ^*#*^			
LDL cholesterol (mg/dL)^**∗**^	111 ± 23.90	109 ± 28.9	95.3 ± 30.8	*0.022*	0.875	0.018	0.095
TG (mg/dL)	58 [48.0; 76]	128 [80.0; 153]	108 [84; 168]	*<0.001*	<0.001	<0.001	0.857
SBP (mmHg)	121 [113; 132]	143 [134; 158]	140 [130; 158]	*<0.001*	<0.001	<0.001	0.665
DBP (mmHg)^**∗**^	74.7 ± 7.95	83.3 ± 10.6	78.1 ± 12.6	*<0.001*	<0.001	0.254	0.061
CRP (mg/L)	0.82 [0.50; 1.96]	1.38 [0.78; 3.65]	4.13 [2.27; 9.80]	*<0.001*	0.099	<0.001	0.001
25D (ng/mL)	25.7 [20.2; 30.4]	28.1 [20.4; 33.0]	12.7 [8.80; 20.7]	*<0.001*	0.556	<0.001	<0.001
VEGF (pg/mL)	233 [112; 372]	398 [230; 599]	373 [219; 507]	*0.002*	0.004	0.013	0.595

^*∗*^Identified variables with normal distribution. Values of these variables are described with mean ± standard deviation. For variables with significant deviations from normal distribution, values are provided as median [IQR]. ^#^Adjusted *p* values in multiple comparisons are not performed for variables with nonsignificant differences according to the overall test (*p* overall). BMI: body mass index; WC: waist circumference; TG: triglycerides; SBP: systolic blood pressure; DBP: diastolic blood pressure; CRP: C-reactive protein; 25D: 25-hydroxyvitamin D; VEGF: Vascular Endothelial Growth Factor.

**Table 2:** Carotid IMT and carotid adventitial VV values in control subjects and patients affected by chronic kidney disease.

		Control (*n* = 65)	CKD 3-4 (*n* = 44)	CKD 5D (*n* = 37)	LRT *p*	p (C versus CKD 3-4)	*p* (C versus 5D)	*p* (CKD 3-4 versus 5D)
A	Right cIMT^**∗**^	0.63 ± 0.11	0.71 ± 0.15	0.78 ± 0.14	<0.001^a^ (0.006^ab^)	0.005^a^ (0.248^ab^)	<0.001^a^ (0.002^ab^)	0.028^a^ (0.043^ab^)
	Left cIMT^**∗**^	0.66 ± 0.13	0.72 ± 0.15	0.79 ± 0.16	<0.001 (0.055^b^)	0.030	<0.001	0.053

B	Right VV	0.59 [0.44; 0.70]	0.69 [0.56; 0.80]	0.62 [0.52; 0.73]	0.011^a^ (0.022^ab^)	0.003^a^ (0.007^ab^)	0.227^a^ (0.295^ab^)	0.147^a^ (0.147^ab^)
	Left VV	0.56 [0.49; 0.66]	0.58 [0.50; 0.68]	0.53 [0.42; 0.66]	0.606^a#^ (0.487^ab^)			

^*∗*^Identified variables with normal distribution. Descriptive values are expressed as mean ± SD or median [IQR] depending on their normal distribution according to Shapiro-Wilks test. ^#^Adjusted *p* values in multiple comparisons are not performed for variables with nonsignificant differences according to the overall test (*p* overall). The column “LRT *p*” refers to the likelihood ratio test *p* value measuring the overall differences among groups by comparing the models with and without group identification. ^a^Box-Cox transformation of the dependent variable to get normally distributed residuals from the multivariate linear regression analysis. ^b^Age adjusted *p* value in linear regression analysis.

**Table 3:** Association between adventitial VV and biochemical and anthropometric variables in CKD patients.

	CKD 3-4				CKD 5D			
	Right VV		Left VV		Right VV		Left VV	
	Correlation	*p* value	Correlation	*p* value	Correlation	*p* value	Correlation	*p* value
Age (years)	0.0666	0.6913	−0.1730	0.3279	−0.0788	0.6790	0.2077	0.2708
WC (cm)	0.1106	0.5084	−0.0870	0.6245	−0.0627	0.7466	−0.1939	0.3064
BMI (Kg/m^2^)	0.1226	0.4635	−0.0212	0.9051	0.2107	0.2636	−0.1618	0.3931
Glucose (mg/dL)	0.2134	0.2183	−0.2012	0.2777	**0.4691**	**0.0089**	0.0726	0.7029
Cholesterol (mg/dL)	0.0507	0.7722	0.1797	0.3335	0.3547	0.0545	−0.2098	0.2658
HDL cholesterol (mg/dL)	−0.2872	0.0943	−0.1220	0.5133	**0.5044**	**0.0073**	0.1116	0.5871
LDL cholesterol (mg/dL)^**∗**^	0.1696	0.3301	0.0217	0.9076	0.2486	0.2111	−0.1562	0.4461
TG (mg/dL)	0.1711	0.3259	0.3021	0.0986	0.0974	0.6088	−0.1953	0.3009
SBP (mmHg)	0.0165	0.9215	−0.3020	0.0826	−0.1072	0.5945	−0.1881	0.3378
DBP (mmHg)^**∗**^	−0.1329	0.4262	−0.2240	0.2029	−0.0542	0.7885	−0.3296	0.0868
CRP (mg/L)	0.2230	0.3169	0.1123	0.6471	−0.0024	0.9898	0.0265	0.8898
Ca (mg/dL)^**∗**^	0.0117	0.9468	0.0306	0.8702	0.0031	0.9869	−**0.4805**	**0.0072**
P (mg/dL)	−0.0686	0.6943	−0.0157	0.9331	−0.2310	0.2193	−0.1963	0.2986
PTH (pg/mL)	−0.3066	0.1649	−0.1738	0.4768	−0.1056	0.6000	−0.2124	0.2767
25D (ng/mL)	0.0902	0.6175	0.0874	0.6519	0.1395	0.4621	0.1600	0.3984
1,25D (pg/mL)	0.0089	0.9656	0.1404	0.5439	−0.2132	0.2667	0.0175	0.9311
Albumin (mg/dL)^**∗**^	−0.0547	0.7586	0.0710	0.7092	0.1052	0.5802	**0.3785**	**0.0392**
Ferritin (mg/dL)	0.0378	0.8680	−0.2115	0.3847	−0.2234	0.2354	−0.0476	0.8096
Hemoglobin (g/dL)^**∗**^	0.0572	0.7518	−0.0715	0.7123	−0.3118	0.0935	0.0661	0.7286
Fibrinogen (g/L)	0.3145	0.0966	−0.0017	0.9934	0.0913	0.6439	0.1483	0.4515
Leukocytes (10^6^/L)	0.2373	0.1835	−0.2228	0.2454	0.1861	0.3429	0.0889	0.6658
VEGF (pg/mL)	0.0645	0.7037	−0.3152	0.0740	0.2779	0.1370	0.2934	0.1224

^*∗*^Identified variables with normal distribution (Pearson's correlation). For variables with significant deviations from normal distribution, Spearman's correlation was applied. Statistically significant *p* values are indicated in bold. WC: waist circumference; BMI: body mass index; TG: triglycerides; SBP: systolic blood pressure; DBP: diastolic blood pressure; CRP: C-reactive protein; Ca: serum calcium; P: serum phosphorus; PTH: parathyroid hormone; 25D: 25-hydroxyvitamin D; 1,25D: 1*α*,25-dihydroxyvitamin D; VEGF: Vascular Endothelial Growth Factor.

**Table 4:** Association between adventitial VV and clinical dichotomous variables in CKD patients.

	Right VV				Left VV			
	CKD 3-4		CKD 5D		CKD 3-4		CKD 5D	
	Median [IQR]	*p* value	Median [IQR]	*p* value	Median [IQR]	*p* value	Median [IQR]	*p* value
Sex								
Man	0.69 [0.58; 0.81]	0.586	0.65 [0.54; 0.74]	0.645	0.60 [0.55; 0.66]	0.275	0.52 [0.44; 0.78]	0.708
Woman	0.68 [0.48; 0.78]		0.61 [0.51; 0.70]		0.56 [0.46; 0.65]		0.55 [0.42; 0.61]	
Diabetes								
No	0.65 [0.53; 0.79]	0.397	0.56 [0.51; 0.69]	**0.038**	0.59 [0.50; 0.66]	0.881	0.52 [0.43; 0.64]	0.604
Yes	0.73 [0.65; 0.88]		0.70 [0.68; 0.75]		0.57 [0.53; 0.65]		0.57 [0.46; 0.65]	
Hypertension								
No	0.86 [0.60; 0.90]	0.188	0.66 [0.55; 0.81]	0.534	0.69 [0.58; 0.79]	0.661	0.50 [0.47; 0.76]	0.604
Yes	0.67 [0.51; 0.78]		0.59 [0.53; 0.71]		0.58 [0.51; 0.65]		0.54 [0.42; 0.64]	
Dyslipidemia								
No	0.66 [0.58; 0.78]	0.921	0.57 [0.54; 0.75]	0.698	0.65 [0.56; 0.74]	0.155	0.55 [0.48; 0.60]	0.860
Yes	0.70 [0.50; 0.81]		0.63 [0.51; 0.69]		0.57 [0.48; 0.61]		0.50 [0.42; 0.80]	
Smoking status								
Nonsmoker	0.70 [0.56; 0.80]	0.062	0.63 [0.52; 0.76]	0.460	0.56 [0.49; 0.61]	0.068	0.55 [0.42; 0.67]	0.450
Former smoker	0.53 [0.46; 0.64]		0.70 [0.54; 0.74]		0.68 [0.62; 0.79]		0.53 [0.48; 0.71]	
Smoker	0.79 [0.71; 0.84]		0.56 [0.53; 0.57]		0.64 [0.59; 0.74]		0.47 [0.44; 0.49]	

Differences in the right or the left adventitial VV distribution depending on sex, diabetes, hypertension, dyslipidemia, and smoking in nondialysis and dialysis patients.

**Table 5:** Clinical and biochemical differences between nondialysis and dialysis CKD patients.

	CKD 3-4 (*n* = 44)	CKD 5D (*n* = 37)	*p* overall
Diabetes			
No	35 (79.5%)	30 (81.1%)	1.000
Yes	9 (20.5%)	7 (18.9%)	
Hypertension			
No	5 (11.4%)	7 (18.9%)	0.522
Yes	39 (88.6%)	30 (81.1%)	
Dyslipidemia			
No	11 (25.0%)	14 (37.8%)	0.315
Yes	33 (75.0%)	23 (62.2%)	
Etiology			
Diabetes	4 (9.09%)	4 (10.8%)	0.296
Glomerular	6 (13.6%)	12 (32.4%)	
Interstitial	9 (20.5%)	9 (24.3%)	
Polycystic	7 (15.9%)	4 (10.8%)	
Vascular	8 (18.2%)	3 (8.11%)	
Unknown	10 (22.7%)	5 (13.5%)	
Plaques			
No	21 (47.7%)	13 (35.1%)	0.359
Yes	23 (52.3%)	24 (64.9%)	
Right plaques			
No	25 (56.8%)	17 (45.9%)	0.452
Yes	19 (43.2%)	20 (54.1%)	
Left plaques			
No	27 (61.4%)	21 (56.8%)	0.847
Yes	17 (38.6 %)	16 (43.2%)	
Smoking			
Nonsmoker	15 (50%)	23 (62.2%)	0.517
Former smoker	7 (23.3%)	8 (21.6%)	
Smoker	8 (26.7%)	6 (16.2%)	
Patients on ESA treatment			
No	42 (95.5%)	5 (14.7%)	**<0.001**
Yes	2 (4.5%)	29 (85.3%)	
NESP dose (*µ*g/kg/week)	0.64 [0.62; 0.66]	0.33 [0.17; 0.57]	0.198
Patients on iron treatment			
No	39 (88.6%)	17 (51.5%)	**0.001**
Yes	5 (11.4%)	16 (48.5%)	
Dose (g/month)	105 [100; 105]	102 [100; 250]	
Ca (mg/dL)^**∗**^	9.22 ± 0.41	8.84 ± 0.56	**0.001**
P (mg/dL)	3.62 [3.22; 4.29]	4.16 [3.71; 5.38]	**<0.001**
PTH (pg/mL)	8.80 [6.80; 13.8]	29.7 [17.1; 47.7]	**<0.001**
1,25D (pg/mL)	38.5 [21.0; 45.5]	5.90 [5.90; 10.2]	**<0.001**
Albumin (mg/dL)^**∗**^	4.46 ± 0.32	4.25 ± 0.32	**0.006**
Ferritin (mg/dL)	156 [59.2; 195]	293 [143; 468]	**0.001**
Hemoglobin (g/dL)^**∗**^	13.8 ± 1.79	12.4 ± 1.15	**<0.001**
Fibrinogen (g/L)	4.25 [3.82; 4.68]	4.40 [4.10; 5.10]	0.232
Leukocytes (10^6^/L)	6.29 [5.32; 8.58]	5.77 [4.46; 6.98]	0.056
REGICORE	4.00 [3.00; 5.50]	2.50 [1.75; 5.25]	0.283
Score	1.00 [1.00; 2.00]	1.00 [0.00; 2.00]	0.374

^**∗**^Identified variables with normal distribution. Values of these variables are described with mean ± standard deviation. For variables with significant deviations from normal distribution, values are provided as median [IQR]. Statistically significant values are indicated in bold. NESP: darbepoetin; Ca: serum calcium; P: serum phosphorus; PTH: parathyroid hormone; 1,25D: 1*α*,25-dihydroxyvitamin D; REGICORE: REgistre GIroní del COR (cardiovascular risk chart).

**Table 6:** Adjusted association between RVV and dialysis in CKD patients.

Coefficients	Estimate	Std. error	Pr (>*t*)
(Intercept)	0.53955	0.14587	0.000623
Hb (g/dL) [11.5, 12.5)	0.11525	0.14998	0.446544
Hb (g/dL) ≥ 12.5	0.36840	0.13735	0.010411
Dialysis	0.17840	0.14382	0.221679
Hb (g/dL) [11.5, 12.5): dialysis	−0.04890	0.17228	0.777940
Hb (g/dL) ≥ 12.5: dialysis	−0.42202	0.15385	0.008912
1,25D (pg/mL) [12.1,66.0]	−0.17625	0.08000	0.033131
P (mg/dL) [4.00, 7.31]	−0.10963	0.06917	0.120476
1,25D (pg/mL) [12.1,66.0]: P (mg/dL) [4.00, 7.31]	0.24083	0.10556	0.027658

Multiple *R*-squared: 0.3592. Hb was recoded into three levels according to values 11.5 and 12.5 based on its relationship with RVV. Levels of 1,25D were recoded into two levels according to their median in the subsample without missing values in the variables of the model. Hb: hemoglobin; 1,25D: 1*α*,25-dihydroxyvitamin D; P: serum phosphorus.
